# Oral Health Disparities in Type 2 Diabetes: Examining the Elevated Risk for Dental Caries—A Comparative Study

**DOI:** 10.3390/dj13060258

**Published:** 2025-06-10

**Authors:** José Frias-Bulhosa, Maria Conceição Manso, Carla Lopes Mota, Paulo Melo

**Affiliations:** 1 Faculty of Dental Medicine, University of Porto, 4200-393 Porto, Portugal; jfrias@ufp.edu.pt (J.F.-B.); paulomelopt@gmail.com (P.M.); 2 FP i3id, FP BHS, Health Sciences Faculty, Fernando Pessoa University, 4200-150 Porto, Portugal; 3 RISE-Health, Faculty of Health Sciences, University Fernando Pessoa, Fernando Pessoa Teaching and Culture Foundation, 4249-004 Porto, Portugal; 4 Associated Laboratory for Green Chemistry (LAQV)/Network of Chemistry and Technology (REQUIMTE), University of Porto, 4050-453 Porto, Portugal; 5 Community Palliative Care Support Team, Unidade Local de Saúde de Gaia e Espinho, 4434-502 Vila Nova de Gaia, Portugal; carlalopesmota@gmail.com; 6 Institute of Public Health, Epidemiology Research Unit (EPI Unit), 4050-600 Porto, Portugal; 7 Laboratory for Integrative and Translational Research in Population Health (ITR), Institute of Public Health, University of Porto, 4050-600 Porto, Portugal

**Keywords:** dental caries, diabetes complications, type 2 diabetes, dental diseases

## Abstract

**Background/Objectives**: Dental caries (DCs) and type 2 diabetes share common risk factors. Dental caries risk in type 2 diabetics (T2DM) shows contradictory results. The aim of this study was to determine if there is a difference in DC prevalence in adults with and without T2DM and whether body mass index (BMI) classes or glycated hemoglobin (HbA1c) levels interfere in that difference. **Methods**: A total of 666 adults (n(T2DM) = 343; n(nT2DM) = 323), from Espinho Primary Health Care Center, were interviewed by calibrated observers. Data from clinical records were collected and oral health status was registered using WHO criteria. Inference analysis was conducted using non-parametric tests (α = 0.05). **Results**: A similar caries prevalence was found between the T2DM (98.2%) and nT2DM (98.8%) groups, with the T2DM group showing significantly higher tooth loss (*p* < 0.001), higher caries experience rerted as mean ± sd (17.7 ± 8.3 vs. 15.9 ± 7.8, *p* = 0.005), fewer decayed teeth (*p* < 0.001) and filled teeth (*p* = 0.016) compared to nT2DM. The most frequently identified comorbidity was hypertension (53.6%). Tobacco use (12.9%) was lower in T2DM (*p* < 0.001). The restorative and treatment indices indicated a significantly higher proportion of use of oral care services (*p* < 0.001) in T2DM individuals. The prevalence of the higher classes of BMI indicative of pre-obesity or obesity shows significant differences (*p* < 0.001). The differences found in the DMFT or each of its components for the prevalence or for the mean in HbA1c control were not statistically significant (*p* = 0.368, and 0.524, respectively). **Conclusions**: Adults with T2DM and higher BMI classes could be associated with a greater prevalence of DCs. The glycemic control of T2DM does not significantly influence DMFT score or each of its components.

## 1. Introduction

Dental caries (DCs) and type 2 diabetes mellitus (T2DM) are significant public health issues globally [1,2]. These conditions often intersect and share common risk factors, with overexposure to refined carbohydrates being a significant contributor to the development of dental caries (DCs) [3,4]. This occurs through the prolonged stay of glucose in saliva, which indirectly enhances the metabolic activity of the oral microflora, alters the dental biofilm, and ultimately promotes the occurrence of DCs [5,6].

Diabetes mellitus is a metabolic disease caused by dietary and metabolic imbalances, characterized by chronic hyperglycemia and disrupted carbohydrate, protein, and fat metabolism [7,8]. It is also associated with increased risk of coronary heart disease, various vascular disorders, and obesity [7,8].

The literature presents a contradictory pattern regarding the relationship between DCs and T2DM, with differing conclusions reported across a scoping review [3], a case-control study [9], and a cross-sectional study [10]. Some research indicates that individuals with T2DM are at a higher risk of developing DCs due to various factors, including poor glycemic control, elevated glycated hemoglobin (HbA1c), altered salivary composition, and an increased presence of cariogenic bacteria [7,11,12,13,14,15,16,17,18,19,20,21,22]. There is moderate certainty that diabetes patients have higher DMF index values compared to non-diabetes patients [2,23]. Conversely, some other studies suggest that there is no clear association between DCs and T2DM [13,15,24,25,26,27,28,29].

Several studies [16,18,30,31,32] have examined the association between glycemic status in T2DM and DCs in adults. There is a tendency for salivary flow to decrease when glycated hemoglobin (HbA1c) values increase, and it is known that T2DM patients present a higher prevalence of xerostomia and urea [33]. Additionally, glucose levels in saliva are significantly higher in diabetics compared to healthy individuals, which may facilitate the proliferation and colonization of pathological bacteria in the oral cavity of diabetic patients [33,34,35].

In the same sense, the body mass index (BMI) was not associated to DCs in several studies with diabetic participants [36,37], although there are some studies [3,38] that indicate this association in a non-diabetic population.

The aim of this study was to determine whether there is a difference in the prevalence and experience of DCs between adults with and without T2DM and to assess whether body mass index (BMI) categories or glycated hemoglobin (HbA1c) levels influence this difference.

## 2. Materials and Methods

A cross-sectional study was conducted among adult volunteers who were randomly invited to participate, including both those with type 2 diabetes mellitus (T2DM) and those without (nT2DM), and who were attending the Family Health Unit in Espinho, a suburban city in northern Portugal.

The study was approved by the Ethical and Clinical Research Committee of Health Regional Administration—North (CES/ARSN n.8/2013).

Two distinct groups were formed. One group included all patients with T2DM who had no other major health problems, while the other group (nT2DM) consisted of healthy volunteers with no history of T2DM or any other significant health issues. Exclusion criteria included individuals younger than 18 years old, those diagnosed with type 1 diabetes or gestational diabetes, and individuals with any physical or mental disorders that would prevent an oral examination.

From a total population of approximately 14,000 individuals registered at USF Espinho, the study sample was determined based on the number of users diagnosed with T2DM (N = 1124), and a control group of users who had attended a medical consultation at USF Espinho but had not been diagnosed with T2DM in the previous three years. The minimum required sample size (n = 656; 328 per group) was calculated using the OpenEpi^®^ software. The parameters used included an expected dental caries prevalence of 65% ± 5%, a minimum detectable difference of 5%, a 95% confidence interval, and a power of 90%. These estimates were based on adult caries prevalence data from the most recent national oral health survey in Portugal (3rd National Epidemiologic Study for Oral Diseases) [39]. A sample of 666 individuals (343 in T2DM group and 323 for nT2DM group) participated in this study.

All participants were interviewed by a family doctor (CLM), and informed consent was requested to be signed twice in independent forms. One form was used for the identification and registration of health data related with T2DM (age, sex, duration of illness, BMI, smoking habits, HbA1c value, retinopathy, nephropathy, neuropathy), and another form concerned the oral cavity observation and registration of oral health indicators. Both registration forms were anonymized, and the researchers completed them during the personal interview.

There was a small number of dropouts (<5 in both groups) after the invitation, and the main reason was related to the availability of time for participants to complete the questionnaire and participate in the oral exam.

The HbA1c, BMI, duration of illness, and evaluation of the presence of hypertension, retinopathy, nephropathy, and neuropathy were obtained by consulting the individual’s electronic or paper clinical process. The clinical analytic data considered was the latest one available from the 12 months prior to the interview.

Participants were initially classified into BMI categories according to the following standard cut-off points: underweight (BMI < 18.5 kg/m^2^), normal weight (BMI 18.5–24.9), overweight or pre-obese (BMI 25–29.9), and obese (BMI ≥ 30). Obesity was further categorized into Class I—moderate obesity (BMI 30–34.9), Class II—severe obesity (BMI 35–39.9), and Class III—very severe or morbid obesity (BMI ≥ 40).

Oral status was recorded using the WHO oral health criteria [40] with the aid of a questionnaire and clinical assessment on DMFT. A single experienced examiner (JFB) who was trained and calibrated performed observation. To assess the consistency between observations, the examiner randomly repeated several clinical observations during the recording phase (Cohen’s kappa of 0.80).

Using the data of decayed, missing, and filled teeth (DMFT), the care index (CI), the restorative index (RI), and the treatment index (TI) were calculated, all expressed as percentages. CI is defined as the number of restored teeth as a fraction of the total number of decayed (DT), missing (MT), and filled (FT) teeth (CI = FT/DMFT × 100); RI is the number of filled teeth (FT) as a fraction of the total number of decayed (DT) and filled (FT) teeth (RI = FT/(DT + FT) × 100); TI indicates the treatment received by an individual and is the number of missing teeth and filled teeth as a fraction of DMFT (TI = (MT + FT)/DMFT × 100) [41].

Statistical analysis was performed using the IBM© SPSS^®^ Statistics v.26 (IBM© Corporation, Chicago, IL, USA). The level of significance was set to 0.05 for all inference situations.

Quantitative variables (age, DC (i.e., DT, MT, FT and DMFT), and HbA1c (%)) were described as mean and standard deviation (sd), while categorical variables (gender, systemic pathologies, oral hygiene, smoking habits) were described as counts and percentages (n, %).

Comparison of mean values between two groups was carried out using the Student’s independent *t*-test for a normal distribution. The Kolmogorov–Smirnov test was used for DC. Mean and standard deviation (sd) were used for describing the other quantitative variables. For DCs, individual median scores and the non-parametric Mann–Whitney test (two independent groups) or the Kruskal–Wallis test (≥3 groups) were used. Comparison of DC scores by BMI groups were performed, including the “low weight” within “normal weight” and the “morbid obese” within the “obese” patients’ data (this decision was based on the highly uneven distribution of participants across the original BMI categories).

The Spearman correlation coefficient was used to test the association between the continuous variables—decayed, missing, or filled teeth, or DMFT—and BMI or glycated hemoglobin (HbA1c, (%)).

The prevalence of DCs, gender, BMI, tobacco consumption, and daily oral hygiene and moment of the day, duration of type 2 diabetes, and systemic pathologies (nephropathy, retinopathy, neuropathy, and dyslipidemia) were compared using the chi-square test.

The confidence intervals (95% CL) for the DC mean value and DC prevalence (%) were calculated using the adjusted Wald method.

## 3. Results

A total of 666 adult participants were successfully recruited for the study, all of whom completed the questionnaire and underwent an oral examination. Although there were a small number of refusals, the main reason was lack of time to participate in both the questionnaire and the oral examination.

The majority of participants were female (56.9%), the mean age was 63.9 ± 12.8 years old, and the prevalence of reported daily oral hygiene was 74.6% (T2DM) vs. 75.5% (nT2DM), with significantly more nT2DM participants brushing at night (*p* = 0.018). The mean DMFT was 17.7 ± 8.3 for T2DM and 15.9 ± 7.8 for nT2DM (*p* = 0.005), and DMFT prevalence was 98.2% for T2DM and 98.8% for nT2DM, not significantly different for both groups (Table 1).

In T2DM patients, the gender ratio was well balanced, but the mean age is slightly higher but without a significant difference (*p* = 0.533). The prevalence of obesity (BMI ≥ 30 kg/m^2^) was significantly higher in individuals with T2DM (*p* < 0.001), whereas the prevalence of pre-obesity (BMI 25.0–29.9 kg/m^2^) and low weight/normal weight (BMI < 25.0 kg/m^2^) categories was significantly greater among nT2DM individuals (*p* = 0.021 and *p* < 0.001, respectively). Among nT2DM patients, there was a higher participation of women (*p* = 0.003).

The results show significant mean differences for DMFT, DT, MT, and FT. The T2DM patients showed significantly higher tooth loss (*p* < 0.001), DMFT (*p* = 0.005) and fewer decayed teeth (*p* < 0.001) and filled teeth (*p* = 0.016) compared to nT2DM patients. The results also show significant differences (*p* < 0.001) between T2DM and nT2DM in CI (15.4% vs. 20.6%), RI (57.3% vs. 53.6%), and TI (88.6% vs. 82.2%).

Table 2 describes disease experience in T2DM patients. The mean duration of disease was 9.6 ± 8.1 years; the most frequent comorbidity of T2DM patients was arterial hypertension (53.6%) and a significantly higher percentage (70%) showed controlled diabetes (*p* < 0.001) with a mean HbA1c for this group of 6.2 ± 0.4.

**Figure 1:**
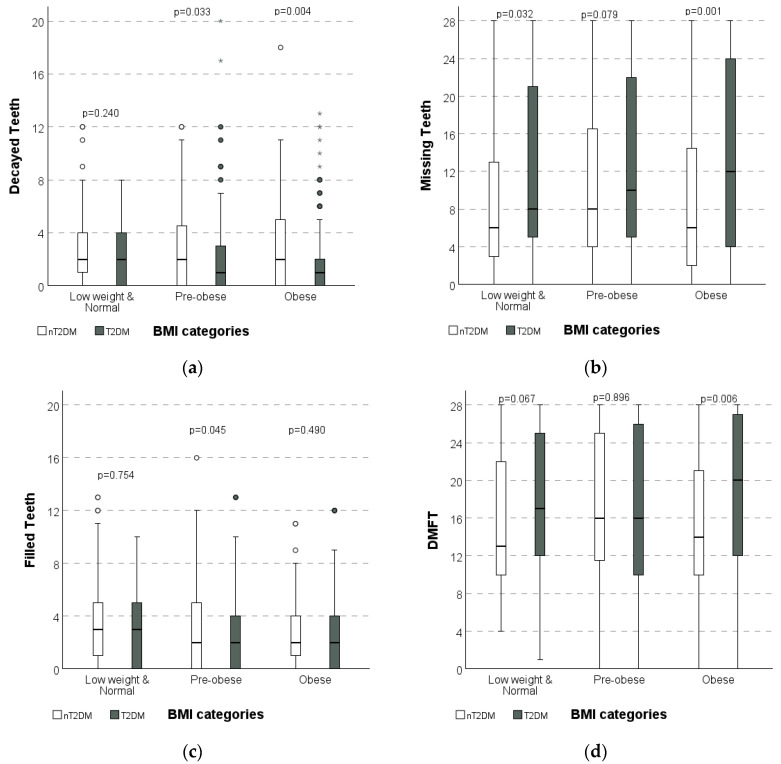
Box plots illustrate the comparison between groups (nT2DM and T2DM) regarding the distribution with BMI categories: (**a**) decayed teeth (DT); (**b**) missing teeth (MT); (**c**) filled teeth (FT); (**d**) decayed, missing, and filled Teeth (DMFT). Dots indicate outlier observations, whereas asterisks indicate extreme outliers.

No significant differences in DT, MT, FT, or DMFT median scores were observed across BMI classes when considered independently. However, significant differences were observed between individuals with and without T2DM in the distribution of median DT (*p* = 0.004), MT (*p* = 0.001), and DMFT (*p* = 0.006) scores when stratified by BMI classification into pre-obese and obese categories (Table 3 and Figure 1a–d).

A similar pattern of no significant association was observed between either caried teeth, lost teeth, filled teeth, or DMFT and BMI (quantitative variables) for either nT2DM (n = 323, r_s_ = −0.017, *p* = 0.756; r_s_ = 0.029, *p* = 0.600; r_s_ = −0.028, *p* = 0.610; r_s_ = 0.035, *p* = 0.528, respectively) or T2DM individuals (n = 342, r_s_ = −0.118, *p* = 0.129; r_s_ = 0.060, *p* = 0.268; r_s_ = −0.006, *p* = 0.911; r_s_ = 0.052, *p* = 0.336, respectively).

**Figure 2:**
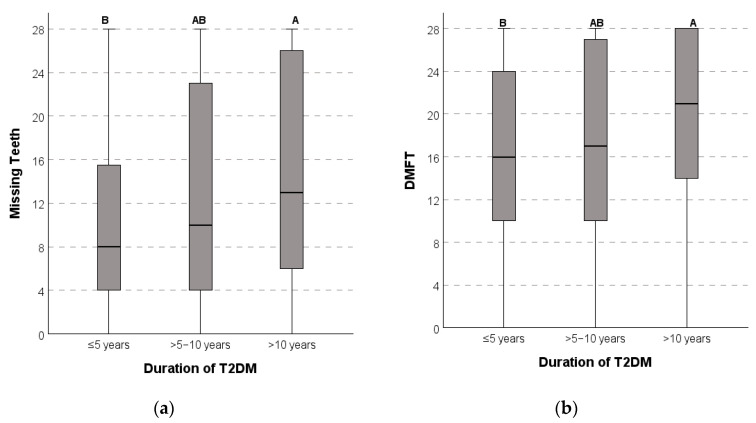
Box plots illustrate the following relationships: (**a**) median missing teeth (MT) scores are significantly higher in the obese BMI category (*p* = 0.014); (**b**) median DMFT scores increase significantly with longer durations of T2DM (*p* = 0.030). In both cases, participants with a T2DM duration greater than 10 years exhibit significantly higher median MT or DMFT scores compared to those with a disease duration of 5 years or less. Letters “A” and “B” in the graphs indicate groups with statistically significant differences in median values, based on Mann–Whitney tests corrected for multiple comparisons after a Kruskal–Wallis test.

Individuals with T2DM diagnosed for more than 10 years showed, compared to those diagnosed with a shorter disease time, significantly higher HbA1c (*p* < 0.001) (Table 2), as well as higher MT (*p* = 0.014) and DMFT (*p* = 0.03) (Table 3 and Figure 2a,b).

For T2DM participants, the analysis using continuous values of either caried teeth, lost teeth, filled teeth, or DMFT and HbA1c (%) also showed no significant association (n = 342, r_s_ = 0.013, *p* = 0.804; r_s_ = −0.015, *p* = 0.784; r_s_ = −0.025, *p* = 0.652; r_s_ = 0.027, *p* = 0.613, respectively).

Although the proportion of individuals who do not smoke was significantly lower among those diagnosed with T2DM (*p* < 0.001; Table 1), it was found that non-tobacco users without T2DM exhibited significantly higher FT scores compared to non-tobacco users with T2DM (3.5 ± 3.5 vs. 2.6 ± 2.4 teeth; *p* = 0.021; Table 3).

## 4. Discussion

The sample comprised a clinical population attending a public primary care center. Historically, this population had limited access to dental care and preventive oral health interventions—according to the results of the third national study on the prevalence of oral diseases in Portugal [39], only 54.7% of adults aged 35–44 and 40.6% of adults aged 65–74 had had a dental consultation in the last year—which could explain the high prevalence of dental caries observed. In 2022, 8.2% of the Portuguese population reported unmet dental care needs, one of the highest rates in the EU [42]. Cost was the main barrier, reflecting limited dental coverage under the national health system. This issue disproportionately affected low-income groups, with 17.5% of the lowest quintile reporting unmet needs, compared to less than 1% in the highest [42]. However, the results of the present study indicated a significant association between DCs and T2DM.

DCs did not show a normal distribution (Kolmogorov–Smirnov test) in both groups. Although median and interquartile range statistics would more precisely describe these variables, the mean and standard deviation (sd) were used to maintain consistency with the central tendency and variability statistics of other quantitative variables, facilitating better comparison with other studies. Despite the reporting of means (sd), the comparison of DC variables was performed using individual median values between categories of the main covariates, using the non-parametric Mann–Whitney test (for two independent groups) or the Kruskal–Wallis test (for three or more groups) (Table 1 and Table 3). For the comparison of DC scores by BMI groups, the “low weight” group was combined with the “normal weight” group, and the “morbidly obese” group was combined with the “obese” group, as these two groups had very small sizes compared to the other BMI groups (Table 3).

The higher participation of women in the nT2DM group (Table 1) may be attributed to the traditional trend of women accessing primary health care. Additionally, the absence of gender differences in the findings suggests that the greater representation of women in the study did not bias the results [43].

Tobacco consumption was statistically lower (*p* < 0.001) among individuals diagnosed with T2DM (Table 1) than in nT2DM. This may be justified by the greater adherence to smoking cessation after the diagnosis of diabetes is established, and this is consistent with Roderick P et al. findings [44].

As reported by Žiūkaitė et al. [45], there seems to be a moderate certainty that the risk of being edentulous for diabetic patients is higher than that for non-diabetic patients. They found an odds ratio of 2.39 (95% CI: 1.73, 3.28, *p* < 0.00001). These data may be related to a higher burden of oral disease among people with T2DM. In this study, it was not possible to evaluate the previous intake of glucose-rich dietary habits and its possible contribution to the simultaneous development of DCs and T2DM.

Our study revealed moderate habits in daily oral hygiene (Table 1) regardless of the combinations used between brushing, flossing, or mouth rinses, with the most frequent moments during the day being performed “in the morning” and “at night”. It has been shown that the performance of oral hygiene “at night” is significantly higher in nT2DM patients (*p* = 0.018). This fact may influence the proliferation and colonization of bacteria in the oral cavity, but the difference found in DC, with a significantly lower prevalence in T2DM vs. nT2DM (62.9% vs. 72.8%), is not explained by oral hygiene habits. Regarding nT2DM, no significant differences were found when compared to the latest Portuguese national oral health study [39]: within the 65–74 age demographic, the prevalence of daily brushing at least once was 78.9% in the national study, whereas it was 63.1% in the present study (*p* = 0.251); likewise, the percentage of individuals who reported brushing before bedtime was 71.3% in the national study compared to 67% in the current study (*p* = 0.797).

The results derived from self-reported data necessitate cautious interpretation, as the presence or absence of teeth may affect hygiene practices; notably, a higher percentage of dentate T2DM patients brush their at night compared to their nT2DM counterparts when accounting for only those with teeth, despite there being a larger number of edentulous individuals in the T2DM group (*p* = 0.018), which underscores the complexity of oral hygiene behaviors in this population (*p* = 0.003). At other moments of the day, there is no significant difference in brushing habits in the two groups.

These results are in agreement with the findings of Chang Y et al.’s [46] study. The daily oral hygiene data from this study are also consistent with the most recent national study on the prevalence of oral diseases in Portugal [39], which encompassed the cohorts delineated by the WHO. In that study, the frequency of tooth brushing reported ≥1x/day for the 65–74 age group was 78.9%, and brushing before going to bed was 71.3%. In this study, the corresponding figures are 63.1% and 67%, respectively.

The caries prevalence of 98.2% for the T2DM group (DMFT mean = 17.7) was substantially higher than some recent studies [7,22,31] found, showing a prevalence of 73.3% (DMFT mean = 2.43), certainly justified by the burden of oral disease in adult Portuguese population. In a case–control study, Bharateesh et al. [47] found that although diabetic patients had comparatively less caries, their periodontal status was impaired and the need for complex treatment was higher in diabetic patients (58%) than in controls (41%).

The DMFT (Table 1) was statistically higher (*p* = 0.005) in T2DM than in the nT2DM, but the same was not confirmed considering the prevalence of DMFT > 0 (*p* = 0.585). Our results on the DMFT and their components were similar for T2DM and nT2DM to other studies [28,30,48].

However, in other studies [32,33,34] the main justification for finding a higher prevalence of dental caries in patients with T2DM was explained as a result of the salivary secretion decrease in diabetics and a pH dropdown.

The results indicate a higher prevalence only in the MT component for T2DM. Regarding the mean values of the individual components, there were significant differences between T2DM and nT2DM for all of them, with the T2DM group showing higher mean values for MT and DMFT in comparison.

This may be due in part to the high MT component of the T2DM group, who most likely submitted to a greater number of surgical procedures due to limited access to restorative dentistry care or periodontal disease; consequently, they showed fewer caries lesions (DT, *p* < 0.001) and also fewer fillings (FT).

Data from the DMFT Index report the sum of untreated lesions (DTs) and proportion of the disease treated by both chirurgical (MT) and restorative (FT) procedures. Whilst the DT category reflects untreated carious lesions, the FT and MT categories represent past carious lesions already solved or sequelae of periodontal disease that can also contribute to the MT value.

It is important to emphasize that the MT burden identified can result not only from the therapeutic processes of caries lesions, but also, and particularly in patients with DM2, from the sequelae of periodontal disease, and that the literature reports this association well [29,49].

The extremely low values of correlation (r_s_ ≈ 0) clinically signify no correlation between either caried teeth, lost teeth, filled teeth, or DMFT and BMI (quantitative variables) for both groups. The same was verified for T2DM participants between caried teeth, lost teeth, filled teeth, or DMFT and HbA1c (%). According to the literature [41], one of the ways to evaluate the impact of caries disease and health care assessments could be performed through the Care Index (CI), the Restorative Index (RI) and Treatment Index (TI), which help dentists to gain a better understanding of the disease experience throughout its life cycle and reflect upon previous caries disease management. These data could provide information on the availability of dental services and inequalities in access to care, as well as some information about the nature of dental care (extraction or restorative procedures) and identifying inequities in care provision.

The CI indicates the coverage of the restorative treatment of carious lesions within a population. The RI does not take into account lost teeth, as it assumes uncertainty as to whether teeth were removed due to caries or other factors. The TI reflects individualized treatment and suggests extractions as one of the treatment solutions for DCs by eliminating potential sources of infection.

Significant differences in CI, RI and TI (Table 1) between T2DM and nT2DM patients (*p* < 0.001) were found, representing real differences in the care provision of DCs among the two groups. In terms of DC burden, T2DM patients have shown a CI of 15.4%, compared to a significantly higher frequency in nT2DM patients (20.6%), whereas RI (57.3% vs. 53.6%) and TI (88.6% vs. 82.2%) were significantly higher in T2DM than nT2DM individuals. This might indicate that individuals with T2DM had a lower proportion of restorative treatment and a higher number of missing teeth compared to nT2DM individuals (Table 1). The justification for this may be that, when accessing oral health care, they present more advanced stages of disease, or that tooth loss in T2DM may also be related with periodontal disease, as is well described in the literature [29,45].

The lower percentage for the CI in both groups (T2DM:15.4% vs. nT2DM:20.6%) could represent general difficulties in the access to preventive or curative care. Knowing that diabetics in Portugal do not have any type of special public oral health care plan, the CI can be a tool to try to understand the dental care provided to these chronic patients. The CI will allow supporting future implementation of policies for the prevention of oral complications and the monitoring of its effects along the time.

Various limit values for HbA1c are given in the literature. For this study, it was assumed that values below 6.5% represent a valid control of T2DM, which was the case in the majority of T2DM patients (70.3%) (Table 2).

The subgroup diagnosed with T2DM for more than 10 years had significantly higher HbA1c levels compared to the two subgroups with shorter time of T2DM (*p* < 0.001), which is consistent with the results of other studies [31,50,51]. However, the prevalence of dental caries or its components showed no significant differences in relation to glycosylated hemoglobin control (Table 3).

We found a significant relationship (*p* < 0.001) between poor glycemic control and the duration of the disease (>10 years) (Table 2). The data indicate that most participants had been diagnosed with diabetes for more than 5 years. It has been noted [50] that individuals often have difficulty adapting to significant behavioral changes in the first few years after a T2DM diagnosis. Prolonged duration of T2DM is associated with poor glycemic control, possibly due to progressive impairment of insulin secretion over time resulting from β-cell failure, making reliance on diet alone or oral antidiabetic agents less effective [50,51,52].

The prevalence of microvascular complications (Table 2) of T2DM, such as nephropathy (7%), retinopathy (11.7%), and neuropathy (7%), did not seem to be associated with caries experience.

A higher number of missing teeth (MT) (*p* = 0.014) and DMFT (*p* = 0.030) was found in individuals with an older T2DM diagnosis (>10 years) compared with those with a more recent diagnosis (Table 3), even more, when the DMFT cumulatively assesses DCs throughout life. These findings are also in line with other studies [7,27,49] reporting that higher caries experience was associated with a longer duration of T2DM.

When trying to understand if BMI interferes with different components of DT, MT, FT and DMFT, no significant differences were found between T2DM and nT2DM individuals. BMI is one of the factors that can express historical exposure to a hypercaloric diet and, thus, an increased risk of caries lesions. The prevalence of pre-obese and obese individuals is higher in individuals with T2DM than in nT2DM (Table 1), who had significantly fewer decayed teeth (DT) than those without T2DM in the same BMI categories. In the pre-obese group, T2DM vs. nT2DM was 2.1 ± 2.8 vs. 2.9 ± 3.1 (*p* = 0.033), and in the obese group, it was 1.9 ± 2.8 vs. 3.0 ± 3.3 (*p* = 0.004). Although high BMI may be associated with a caries-risk diet, in the specific group of patients with DMT2 and high BMI in this study, oral disease appears to progress in such a way that teeth are extracted more frequently (possibly due to difficulties in accessing restorative care, advanced stages of disease, or severe periodontal disease), resulting in a higher number of missing teeth (MT) and consequently a lower number of untreated decayed teeth (DT).

The distribution (Table 3) of different DMFT components according to the BMI classes for the two groups is similar to other distributions in studies on adults [36,37].

The higher BMI categories of the T2DM group shows significantly (*p* = 0.033 and *p* = 0.004) less active caries (DTs) (Table 3) than in the nT2DM group. The MT component in the nT2DM low/normal weight and obese class exhibits significantly less extracted teeth than in T2DM individuals. In the FT component, nT2DM pre-obese individuals have significantly more tooth restorations. For DMFT, significant differences were found only in the T2DM obese group, showing higher values than the controls (*p* < 0.001), as described by Kim [37]. Roderick P [44] and Boyajyan V [2] had reported that tobacco use is a risk factor for dental caries associated with changes in saliva and biofilm adhesion. However, our results (Table 3) show that only nT2DM individuals have significantly more FT (*p* = 0.021), and no statistically significant differences were found in DT and FT components between tobacco users. MT (*p* = 0.017) and DMFT (*p* = 0.014) have significantly higher mean values in T2DM users than in nT2DM individuals or compared to non-users.

Within the limitations of the present study, the results suggest that T2DM patients have a higher caries experience (DMFT) and greater edentulism (MT), even in patients with similar oral hygiene habits compared to the nT2DM group. It will be important for future studies to differentiate the impact that periodontal disease can also have on edentulism in diabetics.

Similarly, a limitation can be identified in the loss of granularity when aggregating the extreme BMI classes, but the option of increasing the size of each subgroup can allow for a more robust analysis. Moreover, this study did not include multivariate analysis to control for potential confounding variables, such as BMI and comorbidities, which may influence oral health outcomes independently of T2DM status. As such, the findings should be interpreted with caution (as the selection of patients was limited to a single health center), particularly regarding the extent to which differences in dental caries prevalence and experience can be attributed solely to the presence of T2DM.

## 5. Conclusions

It must be emphasized that the results of this study are only directly comparable with the results of other studies to a limited extent due to methodological problems in connection with the population size and the selection criteria for T2DM and nT2DM groups.

Although public primary health care centers, similar to the one where this study was conducted, provide care for the majority of diabetic patients in Portugal, the results of this clinical sample should be generalized with caution. Nevertheless, basic oral health care programs for this population could potentially be implemented in such settings.

The T2DM group had more missing teeth and greater oral care, according to the CI, RI, and TI used to measure the level of care, with these indices being complementary to each other. One group may have had more tooth extractions than the other when there is a substantial difference in RI but not in TI, and this may indicate the severity of the caries condition or the favoring of surgical options in therapeutic planning.

Higher HbA1c levels, indicating poorer control of diabetes mellitus, may be associated with a higher prevalence of dental caries and filled teeth, although no significant differences were found. Future research should further explore the importance of HbA1c control and the potential impact of periodontal disease on overall tooth loss.

A program to monitor oral health in patients with T2DM should be carried out at the level of primary health care services, combining not only preventive and monitoring components but also therapeutic intervention, similar to the management and monitoring of other macrovascular and microvascular complications in patients with T2DM.

## Figures and Tables

**Table 1:** Demographic and oral clinic parameters of individuals between groups T2DM and nT2DM.

		ALL n = 666	T2DM n = 343	nT2DM n = 323	*p*
Age, mean ± sd		63.9 ± 12.7	67.8 ± 9.8	59.8 ± 14.2	0.533 **
Gender, n (%)					
Female		379 (56.9)	176 ^b^ (51.3)	203 ^a^ (62.8)	0.003 *
Male		287 (43.1)	167 ^a^ (48.7)	120 ^b^ (37.2)	
BMI, n (%)					
Low weight or normal		178 (26.8)	53 ^b^ (15.5)	125 ^a^ (38.7)	<0.001 *’
Pre-obese		286 (42.9)	163 ^a^ (47.5)	123 ^b^ (38.1)	0.021 *’
Obese		202 (30.3)	127 ^a^ (37.0)	75 ^b^ (23.3)	<0.001 *’
Tobacco consumer		86 (12.9)	24 ^b^ (7.0)	62 ^a^ (19.2)	<0.001 *
Total edentulism		83 (12.5)	54 (15.7)	29 (9)	<0.001 *
Daily oral hygiene (all individuals)		500 (75.1)	256 (74.6)	244 (75.5)	0.787 *
In the morning		406 (61.0)	212 (61.8)	194 (60.1)	0.644 *
After lunch		213 (32)	118 (34.4)	95 (29.4)	0.168 *
At night		449 (67.4)	217 ^b^ (63.3)	232 ^a^ (71.8)	0.018 *
Daily oral hygiene (dentate individuals)		435 (74.7)	225 (76.5)	210 (72.9)	0.316 *
In the morning		367 (63.1)	182 (61.9)	185 (64.2)	0.560 *
After lunch		184 (31.6)	88 (29.9)	96 (33.3)	0.378 *
At night		390 (67)	214 ^a^ (72.8)	176 ^b^ (61.1)	0.003 *
Dental caries					
DT	n (%)	451 (67.7)	215 ^b^ (62.9)	235 ^a^ (72.8)	0.006 *
	95% CL %		57.5–67.6	67.7–77.3	
	mean ± sd		2.0 ^b^ ± 2.7	2.8 ^a^ ± 2.9	<0.001 ***
	95%CL mean:		1.74–2.32	2.52–3.16	
MT	n (%)	618 (92.8)	323 ^a^ (94.7)	293 ^b^ (90.7)	0.046 *
	95% CL %		62.5–72.4	87.0–93.5	
	mean ± sd		13.0 ^a^ ± 9.6	9.8 ^b^ ± 8.9	<0.001 ***
	95%CL mean:		11.94–13.99	8.82–10.77	
FT	n (%)	452 (67.9)	213 ^b^ (62.3)	238 ^a^ (73.7)	0.002 *
	95% CL %		56.9–67.1	68.6–78.2	
	mean ± sd		2.7 ^b^ ± 2.9	3.3 ^a^ ± 3.3	0.016 ***
	95%CL mean:		2.39–3.02	2.93–3.65	
DMFT	n (%)	656 (98.5)	336 (98.2)	319 (98.8)	0.585 *
	95% CL %		95.8–99.1	96.7–99.6	
	mean ± sd		17.7 ^a^ ± 8.3	15.9 ^b^ ± 7.8	0.005 ***
	95%CL mean:		16.82–18.57	15.08–16.78	
Care Index (%)			15.4 ^b^	20.6 ^a^	<0.001 *
95% CL %			14.49–16.31	19.56–21.77	
Restorative Index (%)			57.3 ^a^	53.6 ^b^	<0.001 *
95% CL %			56.02–58.67	52.13–55.13	
Treatment Index (%)			88.6 ^a^	82.2 ^b^	<0.001 *
95% CL %			87.73–89.34	81.09–83.18	

^a,b^—different letters show significant differences in counts according to the chi-square *, binomial *’, or in the mean value according to the Student *t*-test ** or the median value according to the Mann–Whitney test *** (for DM and non-DM groups). 95% CL: 95% confidence limits for the parameter (mean or percentage).

**Table 2:** HbA1c distribution and relationship with clinical characteristics of individuals with T2DM diagnosis.

			DM Controlled	DM Uncontrolled	HbA1c
		n (%)	n (%)	n (%)	Mean ± Sd
	All	343			6.8 ± 1.2
T2DM	Controlled	240 ^a^ (70.0)			6.2 ^b^ ± 0.4
	Uncontrolled	103 ^b^ (30.0)			8.2 ^a^ ± 1.3
*p*		<0.001 *			<0.001 **
Duration of T2DM	≤5 years	120 (35.0)	77 ^a^ (40.4)	23 ^b^ (22.3)	6.6 ^b^ ± 1.2
	>5–10 years	109 (31.8)	85 ^a^ (35.4)	24 ^b^ (23.3)	6.5 ^b^ ± 0.9
	>10 years	114 (33.2)	58 ^b^ (24.2)	56 ^a^ (54.4)	7.3 ^a^ ± 1.4
*p*		0.767 ***	<0.001 ***		<0.001 ****
Hypertension		184 (53.6)			6.9 ± 1.3
Nephropathy		24 (7.0)			6.9 ± 1.6
Retinopathy		40 (11.7)			7.1 ± 1.5
Neuropathy		24 (7.0)			6.7 ± 1.4
Dyslipidemia		299 (87.2)			6.8 ± 1.2

^a,b^—different letters show significant differences in the percentage of cases according to the * binomial test and *** chi-square test, or the mean value according to the ** Student *t* test or **** Tukey post hoc comparison test (after ANOVA).

**Table 3:** Distribution and comparison of the mean and standard deviation of DT, MT, FT, and DMFT according to the BMI classes for T2DM and nT2DM individuals.

		DT		*p* *	MT		*p* *	FT		*p* *	DMFT		*p* *
		T2DM	nT2DM		T2DM	nT2DM		T2DM	nT2DM		T2DM	nT2DM	
Variable	Category	Mean ± sd			Mean ± sd			Mean ± sd			Mean ± sd		
BMI	Low weight Normal	2.2 ± 2.1	2.7 ± 2.5	0.240	12.1 ^A^ ± 9.7	9.2 ^B^ ± 8.9	0.032	3.2 ± 2.9	3.4 ± 3.2	0.754	17.4 ± 7.8	15.4 ± 7.5	0.067
	Pre-obese	2.1 ^B^ ± 2.8	2.9 ^A^ ± 3.1	0.033	12.7 ± 9.5	10.7 ± 9.1	0.079	2.5 ^B^ ± 2.9	3.4 ^A^ ± 3.6	0.045	17.2 ± 8.5	16.9 ± 8.0	0.896
	Obese	1.9 ^B^ ± 2.8	3.0 ^A^ ± 3.3	0.004	13.7 ^A^ ± 9.9	9.3 ^B^ ± 8.7	0.001	2.8 ± 3.0	2.9 ± 2.9	0.490	18.4 ^A^ ± 8.2	15.2 ^B^ ± 7.7	0.006
	*p* **	0.115	0.924		0.565	0.211		0.295	0.639		0.478	0.106	
Tobacco consumer	Yes	2.3 ± 2.8	3.4 ± 3.1	0.090	15.2 ^A^ ± 10.2	9.8 ^B^ ± 9.0	0.017	2.8 ± 3.0	2.6 ^b^ ± 2.4	0.965	20.2 ^A^ ± 7.3	15.7 ^B^ ± 8.1	0.014
	No	2.0 ^B^ ± 2.7	2.7 ^A^ ± 2.9	<0.001	12.8 ^A^ ± 9.6	9.8 ^B^ ± 8.9	<0.001	2.7 ^B^ ± 2.9	3.5 ^Aa^ ± 3.5	0.008	17.5 ^A^ ± 8.3	16.0 ^B^ ± 7.7	0.023
	*p* *	0.669	0.125		0.247	0.968		0.954	0.021		0.124	0.791	
Duration of T2DM	≤5 years	2.3 ± 3.0			11.2 ^b^ ± 9.1			3.2 ± 3.2			16.5 ^b^ ± 8.2		
	>5–10 years	1.8 ± 2.0			13.0 ^ab^ ± 9.8			2.4 ± 2.6			17.2 ^ab^ ± 8.6		
	>10 years	1.9 ± 2.9			14.8 ^a^ ± 9.7			2.5 ± 3.0			19.3 ^a^ ± 7.9		
	*p* **	0.416			0.014			0.168			0.030		
T2DM (HbA1c)	Controlled	1.8 ± 2.3			12.9 ± 9.4			2.8 ± 2.8			17.5 ± 8.1		
	Uncontrolled	2.4 ± 3.4			13.0 ± 10.2			2.6 ± 3.1			18.1 ± 8.7		
	*p* **	0.760			0.833			0.282			0.524		
		Prevalence		*p* ***	Prevalence		*p* ***	Prevalence		*p* ***	DMFT > 0		*p* ***
T2DM (HbA1c)	Controlled	63.7%		0.607	96.2%		0.056	64.6%		0.178	98.8%		0.368
	Uncontrolled	60.8%			91.2%			56.9%			97.1%		

^A,B^—different letters show significant differences for the median values of T2DM and nT2DM groups according to the * Mann–Whitney test; ^a,b^—different letters show significant differences for the median values of the categories of the variables according to the * Mann–Whitney test or the ** Mann–Whitney corrected for multiple comparisons after Kruskal–Wallis. *** chi-square test.

## Data Availability

The data presented in this study are available on request from the corresponding author due to privacy.

## Abbreviations

The following abbreviations are used in this manuscript:

DCs	Dental caries
T2DM	Type 2 diabetes mellitus
nT2DM	Non-type 2 diabetes mellitus
BMI	Body mass index
HbA1c	Glycated hemoglobin
DT	Decayed teeth
MT	Missing teeth
FT	Filled teeth
DMFT	Decayed, Missing, and Filled Teeth Index
CI	Care Index
RI	Restorative Index
TI	Treatment Index
